# Stevens-Johnson Syndrome following Failure of Genetic Screening prior to Carbamazepine Prescription

**DOI:** 10.1155/2017/4201357

**Published:** 2017-04-03

**Authors:** Suraya Ahmad Nasir, Huann Lan Tan, Hui Jan Tan, Haizal Mohd Hussaini, Roszalina Ramli

**Affiliations:** ^1^Department of Oral & Maxillofacial Surgery, Faculty of Dentistry, UKM and UKM Medical Centre, Kuala Lumpur, Malaysia; ^2^Department of Medicine, Faculty of Medicine, UKM Medical Centre, Kuala Lumpur, Malaysia; ^3^Department of Oral Diagnostic and Surgical Sciences, Faculty of Dentistry, University of Otago, Dunedin, New Zealand

## Abstract

Failure to screen susceptible individuals for human leucocyte allele B∗1502 leads to the onset of Stevens-Johnson syndrome (SJS). We report a case of a 27-year-old Malay female who was treated with carbamazepine following the diagnosis of trigeminal neuralgia without a genetic screening. She was prescribed 150 mg of carbamazepine initially and the dose was increased to 300 mg following the initial dose. A sudden development of skin and mucous membrane ulcers was observed and this warranted immediate hospitalization. A diagnosis of SJS was made and she was treated immediately with intravenous corticosteroids. Genetic screening prior to carbamazepine prescription is essential especially in susceptible populations.

## 1. Introduction

Carbamazepine (CBZ) remains the first-line therapy for trigeminal neuralgia (TGN) [1, 2]. CBZ which is a dibenzazepine derivative (carboxamides) is a popular drug due to its effectiveness as well as its low cost [2, 3]. However, its side effect can be very devastating. One of the most severe side effects of CBZ is Stevens-Johnson syndrome (SJS). SJS is a rare, life threatening, immune complex hypersensitivity disorder involving the skin and mucous membrane.

The occurrence of SJS is high following intake of CBZ among susceptible Asian people due to the presence of human leucocyte allele B∗1502 (HLA-B∗15:02) [3]. In 2007, the US Food and Drug Administration (FDA) postmarketing adverse events reported that Asian people are highly susceptible to SJS/toxic epidermal necrolysis (TEN) compared to Caucasians and issued the FDA Alert [4].

However, not all health practitioners are aware of this warning. Bell et al. (2013) showed that many of the practicing neurologists still failed to perform pharmacogenomic screening prior to CBZ prescription [5].

We report a case of TGN who was treated with CBZ and developed SJS after a month. Following this case presentation, prevention for recurrent of SJS is discussed.

## 2. Case Report

A 27-year-old Malay lady was first seen in October 2014 in the Oral Medicine Clinic of Universiti Kebangsaan Malaysia Medical Centre (UKMMC), Kuala Lumpur, Malaysia. She complained of sharp electric-shock-like pain over the left upper gingivae for the past one year and the pain was aggravated during tooth brushing. Clinical and radiographic examinations revealed no possible source of odontogenic pathology or infection that could contribute to the pain. Based on the clinical findings, she was diagnosed with trigeminal neuralgia and was prescribed 50 mg CBZ, once in the morning and 100 mg once at night. HLA-B∗15:02 was not performed prior to this prescription.

A week later, her CBZ dosage was increased to 300 mg, once daily, due to poorly controlled neuropathic pain. The facial pain was controlled after this dosage. She denied episodes of dizziness, nausea and constipation following the new dosage.

Unfortunately, five days following the new CBZ regimen, she developed blistering rashes in her vagina and feet. She complained of fever and headache a day before the rashes developed. Clinically, she presented with urticaria involving both her upper and lower limbs and face, oral ulcerations, and conjunctivitis of both eyes. The total white blood cells counts, liver enzymes, and creatinine levels were not raised except for C-reactive protein, which could be associated with the acute inflammation. No further test was performed to determine the cause of allergic reactions as the patient was not exposed to other drugs or allergens prior to the prescription of CBZ and her past medical history was unremarkable. Thus she was diagnosed with SJS secondary to CBZ.

She was immediately treated with intravenous hydrocortisone 50 mg followed by 100 mg three times daily. A combination of analgesia and antiseptic mouthwash (magnesium trisilicate; diphenhydramine; and lignocaine) and 5 mL nystatin 100,000 IU/mL was prescribed for five times daily to ease and treat the painful oral ulcers.

Betamethasone 17 valerate 1 : 4 cream and an aqueous cream were applied twice daily over her skin lesion except the face. Potassium permanganate 1 : 20,000 (5%) 5 mL solution was used during bath, once daily. Her eyes symptom was controlled with sodium hyaluronate 0.18% eye drop, applied twelve times in a day, and Maxitrol eye ointment applied three times daily.

**Figure 1:**
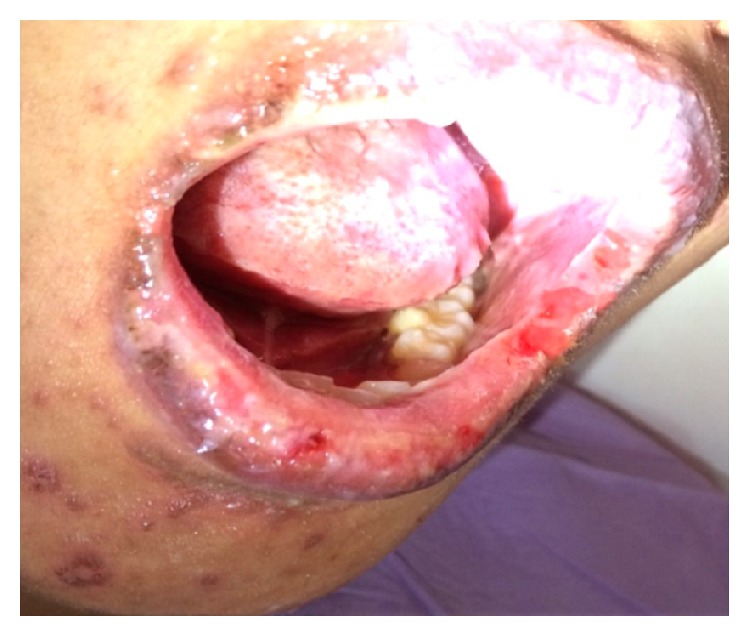
Clinical photograph showing multiple ulceration in the mouth, crusting of the lips, and vesicles and papules of the skin.

The urticaria further progressed into maculopapular rashes and became more significant on the second day of hospitalization, with target lesions on her face and limbs. Similarly, the oral ulcerations became worse (Figure 1).

**Figure 2:**
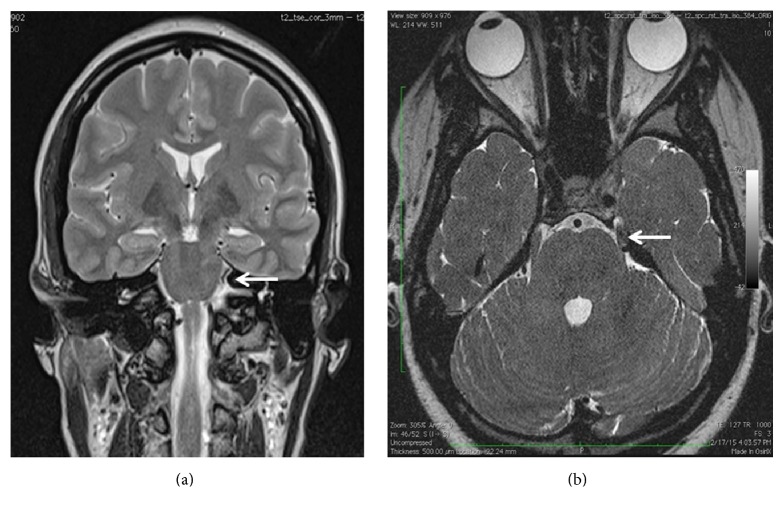
(a) “Coronal view” and (b) “axial view” of the brain showing impingement of cerebellar vein onto the left trigeminal nerve.

The acute phase of inflammation started to resolve on the fourth day of hospitalization and she was discharged home by the eighth day. Brain magnetic resonance imaging (MRI) was performed to exclude nerve compression or multiple sclerosis. The MRI revealed a prominent cerebellar vein impinging on the lateral aspect of the left trigeminal nerve (Figures 2(a) and 2(b)).

The precise location was in the entrance of the nerve to the left Meckel's cave, before the vein drains into the left superior petrosal sinus. This finding correlated with her clinical reported symptoms of TN.

During hospitalization, the patient and her family were counseled by the medical team about the genetic predilection for SJS following CBZ intake, that is, HLA-B∗15:02. CBZ was discontinued completely and the patient was advised against its prescription ever. She was prescribed the allergic card by the hospital pharmacy for this purpose.

A week after her being discharged, she was prescribed with gabapentin 300 mg once daily by the neurologist. Surgical decompression was discussed; however the patient and her family disagreed with this option. The patient was very traumatized with this episode of hospitalization that she refused to take the gabapentin. She also discharged herself from our care before we could proceed with the genetic testing to confirm the presence of HLA-B∗15:02.

## 3. Discussion

The prevalence of HLA-B∗15:02 allele among Asian population is higher compared to the Caucasian population and this was shown to be from 10- to 25-fold higher [6, 7]. Such strong associations had been validated in many populations, especially in the Southeast Asia countries [3, 6, 8, 9]. Various ethnicity has been reported with HLA:B∗15:02 and CBZ-SJS within Southeast Asia: Malaysian multiethnic groups, that is, the Malays, the Chinese, and the Indians [10], the Thai [6], the Singaporean Chinese and Malays [11], and the Vietnamese [12].

Mortality rate of SJS was reported to be from 10% to 40% [13]. SJS has prodromal symptoms and signs such as fever, cough, sore throat, headache, vomiting, myalgia, polyarthralgia, diarrhoea, and lethargy [14]. The reported patient had typical SJS with some of the above prodromal symptoms prior to the mucocutaneous ulcerative lesions.

Trigeminal neuralgia (TGN) pain is often intolerable and interferes with the patients' daily activities. TGN patients were shown to have higher level of anxiety and depression compared to those with atypical pain [15]. Due to the state of pain and level of anxiety, clinicians often rush to treat them. Two lessons learnt from the present case were as follows:

- Brain MRI must be arranged early for young patients suspected with TGN prior to drug prescription. This is to rule out possibility of multiple sclerosis or other structural abnormality or pathology, that is, nerve impingement.
- Genetic screening for HLA-B∗15:02 must be performed prior to prescription of carbamazepine if pharmacological method is the option of treatment.

Although we were unable to confirm the presence of HLA-B∗15:02 in this patient, it was very likely that this patient had CBZ-SJS due to this allele based on her ethnic factor. This was based on Chang et al. (2011) who showed strong association between HLA-B∗15:02 and CBZ-SJS/TENS in Malaysian Malay population [10].

We wish to highlight the pretreatment screening, monitoring regime, and prevention for recurrent SJS for all dental practitioners involved in the prescription of carbamazepine.

### 3.1. Pretreatment Screening

Almost all practicing specialists are familiar with the blood investigations for monitoring the side effects of carbamazepine. However, many are still clueless about the baseline assessment for genetic predilection towards SJS. It was shown that 20% of American neurologists were not aware of antiepileptic safety risks which include haplotype screening in patients of Asian descent prior to starting carbamazepine [5]. In addition, many neurologists, despite recognizing the hypersensitivity reaction, still failed to screen their patients [5].

We anticipate that many other practitioners in various specialties could have the same attitude of unawareness or failure to screen their patients despite the knowledge.

**Figure 3:**
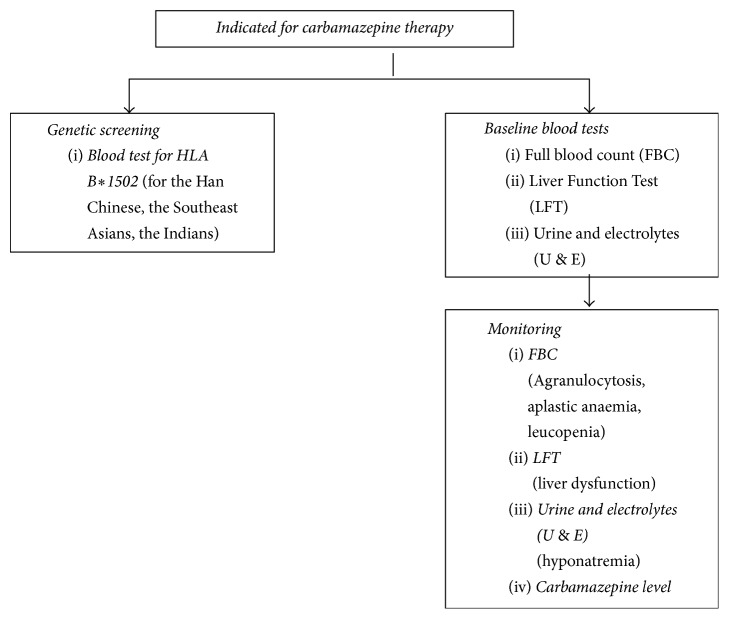
The figure shows the essential blood tests to be performed at the initial stage prior to prescription and following administration of carbamazepine.

Figure 3 summarizes the essential blood investigation prior to and during carbamazepine therapy.

Following the reports on HLA-B∗1502 and SJS, another allele, HLA-A∗31:01, was discovered and was shown to be associated with various hypersensitivity reactions following carbamazepine intake among the European ancestry [16], the Japanese [17], and the Han Chinese [18]. Genin et al. (2014) in their international study showed that HLA-A∗31:01 is a specific predictor for carbamazepine-drug reaction with eosinophilia and systemic symptoms (CBZ-DRESS) but not for CBZ-SJS/TEN [19].

### 3.2. Prevention for Recurrent SJS

The symptoms for carbamazepine-induced SJS usually occur within the first three months following prescription [20]. In addition, in patients who previously have been prescribed carbamazepine for the duration of less than three months, the risk of a SJS occurring in the next prescription is still present even if there was no symptom during the previous administration [20]. These patients require a genetic screening prior to CBZ prescription [20].

For Asian patients who will be prescribed other TGN drugs, that is, phenytoin or oxcarbazepine, genetic screening for HLA-B∗15:02 is also recommended as they have been shown to cause SJS/TEN [21, 22].

Patients who developed SJS have an increased risk for another episode of SJS [23].

Patients who have had SJS in the past should wear a medical bracelet to indicate the drug hypersensitivity.

## 4. Conclusion

Genetic screening particularly in Asian descent must be performed to avoid development of SJS.
